# Characterizing sustained social anxiety in individuals at clinical high risk for psychosis: trajectory, risk factors, and functional outcomes

**DOI:** 10.1017/S0033291722000277

**Published:** 2023-06

**Authors:** Wisteria Deng, Jean Addington, Carrie E. Bearden, Kristin S. Cadenhead, Barbara A. Cornblatt, Daniel H. Mathalon, Diana O. Perkins, Larry J. Seidman, Ming T. Tsuang, Scott W. Woods, Elaine F. Walker, Tyrone D. Cannon

**Affiliations:** 1 Department of Psychology, Yale University, New Haven, CT, USA; 2 Department of Psychiatry, Hotchkiss Brain Institute, Calgary, Canada; 3 Department of Psychiatry and Biobehavioral Sciences and Psychology, UCLA, Los Angeles, USA; 4 Department of Psychiatry, UCSD, San Diego, USA; 5 Department of Psychiatry, Zucker Hillside Hospital, Queens, USA; 6 Department of Psychiatry, UCSF, SFVA Medical Center, San Francisco, USA; 7 Department of Psychiatry, University of North Carolina, Chapel Hill, USA; 8 Department of Psychiatry, Harvard Medical School, Boston, USA; 9 Massachusetts General Hospital, Boston, USA; 10 Department of Psychiatry, Yale University, New Haven, CT, USA; 11 Department of Psychology and Psychiatry, Emory University, Atlanta, USA

**Keywords:** Comorbidity, covariant trajectories, outcome studies, polygenic risk, stress exposure

## Abstract

**Background:**

While comorbidity of clinical high-risk for psychosis (CHR-P) status and social anxiety is well-established, it remains unclear how social anxiety and positive symptoms covary over time in this population. The present study aimed to determine whether there are more than one covariant trajectory of social anxiety and positive symptoms in the North American Prodrome Longitudinal Study cohort (NAPLS 2) and, if so, to test whether the different trajectory subgroups differ in terms of genetic and environmental risk factors for psychotic disorders and general functional outcome.

**Methods:**

In total, 764 CHR individuals were evaluated at baseline for social anxiety and psychosis risk symptom severity and followed up every 6 months for 2 years. Application of group-based multi-trajectory modeling discerned three subgroups based on the covariant trajectories of social anxiety and positive symptoms over 2 years.

**Results:**

One of the subgroups showed sustained social anxiety over time despite moderate recovery in positive symptoms, while the other two showed recovery of social anxiety below clinically significant thresholds, along with modest to moderate recovery in positive symptom severity. The trajectory group with sustained social anxiety had poorer long-term global functional outcomes than the other trajectory groups. In addition, compared with the other two trajectory groups, membership in the group with sustained social anxiety was predicted by higher levels of polygenic risk for schizophrenia and environmental stress exposures.

**Conclusions:**

Together, these analyses indicate differential relevance of sustained *v.* remitting social anxiety symptoms in the CHR-P population, which in turn may carry implications for differential intervention strategies.

Although comorbidity of clinical high-risk for psychosis (CHR-P) status and anxiety symptoms is well established (McAusland et al., 2017), the reasons for this comorbidity are not yet clear. In cross-sectional studies, social anxiety symptoms have been found to be correlated with more severe attenuated psychotic symptoms (McAusland et al., 2017) as well as with poorer social functioning (Fulford et al., 2013; Kuhney et al., 2021). The fact that psychotic symptoms tend to be episodic while social functional deficits tend to be more stable raises the possibility that social anxiety may have different longitudinal trajectories in different subsets of the CHR-P population. Such a pattern, if confirmed, could in turn have differential implications for prediction and treatment/prevention in the subsets.

As a situational symptom, comorbid social anxiety may follow a similar course to the trajectory of positive symptoms (Michail & Birchwood, 2009). Both anxiety and psychosis, especially persecutory beliefs, share similar underlying mechanisms such as an elevated threat anticipation and perception (Michail & Birchwood, 2009). The two symptom dimensions may feed into each other and develop (and subside) concurrently. Situational anxiety may increase the attentional bias to threat, setting the stage for positive symptoms (Freeman & Fowler, 2009), whereas the worsening of positive symptoms may be accompanied by distress in social situations, in turn exacerbating social anxiety (Chang et al., 2021). On the other hand, as a hallmark symptom of schizotypal personality disorder, social anxiety has also been conceptualized as a stable trait indicative of an enduring vulnerability to psychosis (Fonseca-Pedrero et al., 2018; Salokangas et al., 2013). Because stable vulnerability indicators, in turn, tend to be associated with poorer functional outcomes among those with and at risk for psychotic disorders (Rosell, Futterman, McMaster, & Siever, 2014), the presence of a sustained social anxiety symptoms over time may be indicative of a distinctive subgroup of CHR-P cases with respect to risk factors for schizophrenia spectrum disorders and long-term outcomes.

Whether there are distinctive subgroups of CHR individuals with sustained *v.* situational social anxiety symptoms is an open question which cannot be answered using *cross-sectional* research designs or statistical approaches. Rather, *longitudinal* data collection and statistical analysis methods are needed to address these questions. The present study employed group-based multi-trajectory modeling to discern whether different subsets of CHR-P cases from the North American Prodrome Longitudinal Study (NAPLS 2) (Addington et al., 2015) cohort show different patterns of social anxiety and positive symptoms over time. Group-based multi-trajectory modeling is a descriptive data analysis technique for ascertaining covariant patterns of change over time on two or more phenotypic indicators simultaneously. Although this method is sensitive to detecting subgroups with different symptom trajectories, determining whether such trajectory differences are themselves meaningful from an etiological and/or prognostic standpoint is generally considered an important additional step in validation. Here we assessed two major classes of etiologic influence (polygenic risk for schizophrenia and exposure to environmental stressors), as well as general functioning at 2-year follow-up, to determine whether the ascertained trajectory subgroups were distinctive from each other in these respects.

## Methods

### Participants

The participants were recruited as part of the NAPLS 2 study (Addington et al., 2015) which was an eight-site consortium study aiming to investigate predictors and mechanisms of conversion to psychosis. The overall sample consisted of 764 clinical high risk (CHR) participants (436 males, 328 females; ages 12–35) who met the Criteria of Psychosis-Risk Syndromes (COPS) as determined by the Structured Interview for Psychosis-Risk Syndromes (SIPS) (McGlashan, Walsh, & Woods, 2010). Exclusion criteria included any current or lifetime Axis I psychotic disorder (including affective psychoses), any clinically significant developmental or neurological disorder, and current drug or alcohol dependence.

### Measures

#### Symptom measures

*Structured Interview for Prodromal Risk Syndromes* (*SIPS*): The SIPS (McGlashan et al., 2010) was used to determine whether an individual met criteria for a CHR-P syndrome. The Scale of Psychosis-Risk Symptoms (SOPS), used to rate the severity of symptoms, consists of 19 items in four symptom domains: positive, negative, general, and disorganized. The five positive items are unusual thought content, suspiciousness, grandiosity, perceptual abnormalities, and disorganized communication, each rated on a score of 0 (none to minimal) to 6 (present and psychotic in intensity), with ratings of 3–5 representing attenuated (i.e. below diagnostic threshold) psychosis. The five symptom ratings were summed to form a measure of overall positive symptom severity, with a possible range of 0–25, and at least one item scoring higher than or equal to 3 at baseline. The positive symptom domain has a high median reliability of 0.88 across several existing studies (Woods, Walsh, Powers, & McGlashan, 2019).

*Social interaction anxiety questionnaire* (*SIAS*): The SIAS is a self-report questionnaire widely used to measure fears of general social interaction. The SIAS consists of 20 items rated on a five-point Likert scale (0–4), with total scores ranging from 0 (least anxiety) to 80 (most anxiety) (Kupper & Denollet, 2012). A total score of 36 has been used as an empirical threshold for clinical significance (Peters, 2000). The SIAS has a good internal consistency (*α* = 0.89) and test-retest reliability (*r*s of 0.92) (Fergus, Valentiner, Kim, & McGrath, 2014). The SIAS items capture the general experience of anxiety (in forms of distress and worry) in social situations. The measure does not include a temporal anchor, nor does it evaluate behavioral avoidance prompted by the anxiety. As such, the SIAS reflects a generalized, but not necessarily performance-related, social anxiety.

#### Outcome measure

*Global Assessment of Functioning* (*GAF*): As an overall measure of an individual's state of wellbeing, the GAF captures the psychological, social, and occupational aspects of functioning. The GAF score ranges from 100, positive mental health to 0, severe psychopathology and functional disability. The GAF is widely used as a transdiagnostic and multidimensional measure for global functional outcomes (Startup, Jackson, & Bendix, 2002). Our use of the GAF is consistent with its original purpose as a general measure of functioning encompassing both symptom-related distress and social/role functioning dimensions (Pedersen & Karterud, 2012). The GAF has previously been shown to be reliable and valid in studies of patients with psychotic disorders, with interrater reliability (intraclass correlations) ranging from 0.89 to 0.95 (Startup et al., 2002) and with GAF ratings correlating highly with external measures of work and school-related problems and symptom-related distress (Schwartz, 2007; Startup et al., 2002). The GAF provides a reliable and valid global outcome metric that is quantitative rather than qualitative (diagnostic) in nature, encompassing a full range of outcomes related to psychiatric illnesses.

#### Predictor measures

To generate a holistic representation of CHR individuals' experience of environmental stress, three variables of interest were selected reflecting stressors at different time periods in an individual's life (i.e. early life trauma, history of stressful life events, and recent stressors), and a composite baseline environmental stress score was created by taking a sum of the three standardized scores from these three measures, as detailed below.

*Childhood Trauma and Abuse Scale*: Childhood trauma captures an individual's experience of stressors during early developmental stages (Stowkowy et al., 2016). Participants completed the Childhood Trauma and Abuse Scale (Janssen et al., 2004), a semi-structured interview examining experiences of physical, sexual, and psychological abuse, and emotional neglect, occurring prior to age 16 (Addington et al., 2013). Each form of trauma was scored with a binary variable as absent/present, and a total score generated from the sum.

*Psychiatric Epidemiology Research Interview Life Events Scale* (*LES*): Representing the general exposure to environmental stress through later life stages, life events were assessed by modifying the LES to exclude events that were less relevant to youth (e.g. divorce and financial losses) (Dohrenwend, Krasnoff, Askenasy, & Dohrenwend, 1978; Trotman et al., 2014). The modified version of the LES included 59 items pertaining to significant events or life changes that could conceivably be experienced at any of the ages included in the study sample. Interviewers recorded how often each of the 59 events had occurred in the participant's lifetime and the associated level of distress (scored on a seven-point scale: 1 ‘occurred but was not stressful’ to 7 ‘caused me to panic’). Sum of distress was calculated by taking a sum of the amount of distress reported across various life events.

*Daily Stress Inventory* (*DSI*): Daily stressor score provides a momentary assessment of the ongoing distress experienced by an individual. The DSI is a 58-item measure of common daily hassles occurring within the past 24 h (Brantley, Waggoner, Jones, & Rappaport, 1987). Examples of such items include being ‘interrupted during task/activity’ and ‘criticized or verbally attacked’. Participants indicated if the event occurred and rated the amount of distress associated with each endorsed daily hassle on the same seven-point Likert scale, with the total score representing the sum of the stress ratings, as described above.

*Polygenic Risk Score* (*PRS*): Rutgers University's RUCDR Repository and the University of North Carolina's Genomics Core sent DNA extracted from blood to the Broad Institute for analysis with the Illumina PsychArray, version 2, following standard protocols (Perkins et al., 2020). Analysis of raw data followed the RICOPILI (Rapid Imputation and Computational Pipeline for Genome-Wide Association Studies) pipeline (Lam et al., 2020). Imputation used IMPUTE2 and the 1000 Genomes Project phase 1 reference panel. The Broad Institute provided both hard-call data and raw dosage data for further analyses. Further quality control included determination of cryptic relatedness with the KING software package (Manichaikul et al., 2010). We calculated the PRS from the schizophrenia GWAS results from the Psychiatric Genomics Consortium (PGC) (Schizophrenia Working Group of the Psychiatric Genomics, 2014). Based on the raw summary statistics, the PGC provides a list of linkage-disequilibrium pruned SNP association statistics (https://www.med.unc.edu/pgc/). The PRS is a sum of the number of reference alleles weighted by the natural logarithm of the published odds ratio. We included imputed SNPs with INFO score >0.8 and a reported *p* value ⩽0.05 (Schizophrenia Working Group of the Psychiatric Genomics, 2014). The PRS was the residual from the linear regression model of the first 10 principal components projected to the 1000 Genomes Project cohort for non-Europeans and the residual from the linear regression model of the within-European first 10 principal components for Europeans. Further details about the PRS extraction procedure and scoring methods can be found in Perkins et al. (2020).

### Procedures

The NAPLS-2 study was approved by the Institutional Review Boards of all eight participating sites. Informed consent was obtained from those who met the criteria and voluntarily enrolled. Parental consent was obtained from parents/guardians of minors. Participants were assigned to an experienced research clinician as the rater for the SIPS. All raters demonstrated competency on the gold standard post-training agreement that determines a psychosis-risk diagnosis (*κ* ⩾ 0.90) (Addington et al., 2017). Following the initial assessment phase, follow-up assessments were done every 6 months for clinical interviews and symptom measures for up to 2 years (see online Supplementary Table S1). With an attrition rate comparable to other studies of CHR-P samples (Osborne & Mittal, 2019), this study observed that those who were evaluated at 6-, 12-, and 18-month follow-up had significantly less severe positive symptoms at baseline than those who were lost to follow-up (Table 1). While the differential attrition limits the external validity for evaluating symptoms at these timepoints, there were no statistically significant differences in demographic variables and baseline symptom scores between those who were evaluated at 24-month *v.* those lost to follow-up. Thus, differential attrition does not appear likely to explain or limit the generalizability of our findings related to global functional outcomes at 24-month follow-up, at which point the demographic variables and baseline scores of study variables matched between those who were followed *v.* lost to follow-up. In addition, while the differential attrition may have biasing effects on the analyses, it is important to note that a primary reason of the dropouts, especially at these early follow-up timepoints, is conversion to psychosis, an outcome preordained by the CHR-P sample.

**Table 1.** Baseline characteristics for respondents and drop-outs at 6-, 12-, 18-, and 24-month follow-up

	6-month			12-month		
	Respondents *n* = 511	Lost to follow-up *n* = 253	Test statistic	Respondents *n* = 413	Lost to follow-up *n* = 351	Test statistic
	Frequency (%)		χ^2^	Frequency (%)		χ^2^
Sex (female)	215 (42.1)	113 (44.7)	0.46	172 (41.6)	156 (44.4)	0.61
	Mean (*s.d.*)		*t*	Mean (*s.d.*)		*t*
Age (years)	18.45 (*4.32*)	18.61 (*4.05*)	−0.52	18.66 (*4.34*)	18.32 (*4.10*)	1.13
Baseline positive symptoms	11.64 (*3.85*)	12.47 (*3.69*)	−2.85*	11.61 (*3.90*)	12.25 (*3.70*)	−2.35*
Baseline anxiety symptoms	30.85 (*17.45*)	30.68 (*17.35*)	0.11	31.47 (*17.44*)	29.90 (*17.34*)	1.17

	18-month			24-month		
	Respondents *n* = 275	Lost to follow-up *n* = 489	Test statistic	Respondents *n* = 279	Lost to follow-up *n* = 485	Test statistic
	Frequency (%)		χ^2^	Frequency (%)		χ^2^
Sex (female)	122 (44.4)	206 (42.1)	0.36	124 (44.4)	204 (42.1)	0.41
	Mean (*s.d.*)		*t*	Mean (*s.d.*)		*t*
Age (years)	18.43 (*4.39*)	18.54 (*4.15*)	−0.37	18.77 (*4.36*)	18.35 (*4.15*)	1.34
Baseline positive symptoms	11.54 (*3.90*)	12.12 (*3.76*)	−2.01*	11.69 (*4.11*)	12.04 (*3.64*)	−1.20
Baseline anxiety symptoms	31.68 (*17.51*)	30.25 (*17.34*)	1.05	32.00 (*17.35*)	30.05 (*17.42*)	1.44

### Statistical analysis

#### Discovery of social anxiety and positive symptoms multi-trajectory groups

Analyses were conducted in STATA, version 17, using the TRAJ procedure (Jones & Nagin, 2013). We used group-based multi-trajectory modeling, a form of latent growth class analysis, to identify common trajectories of positive symptoms and social anxiety in the NAPLS-2 cohort. This method is used to approximate the true distribution of trajectories by discovering subgroups of individuals who follow similar patterns of change across time on multiple variables (Allswede et al., 2019). Specifically, models simultaneously considered scores for positive symptom severity and social anxiety at each visit when constructing models and assigning group membership. Model comparison began with the estimation of one class and increased in the number of classes until model fit indices did not meet recommended thresholds or identify theoretically meaningful trajectories. Following standard guidelines for this approach, we examined the following fit indices: lower absolute value Bayesian information criteria and log likelihood relative to the previous model, posterior probability of assignment to groups >0.7, group size >5%, and odds of correct classification >5.

#### Outcomes and predictors for group memberships

Our hypotheses focused principally on whether continuity *v.* recovery in co-morbid social anxiety symptoms among CHR-P individuals represents a meaningful distinction with respect to long-term global outcome and risk factors for schizophrenia spectrum disorders. Global functioning ability was used for validating differences in outcome across the derived trajectory groups. GAF score at 24 months following the baseline assessment was compared across groups with different symptom trajectories over five assessment points (i.e. baseline, 6, 12, 18, and 24 months).

Logistic regression analysis was used to investigate potential differences in etiologic predictors according to ascertained trajectory group, treating environmental stress composite score, PRS, and the interaction of the two as predictors. A binary outcome variable was used as the dependent variable, coded as either being allocated in the trajectory group with consistently high levels of social anxiety or not. These analyses were performed using SPSS software version 26.0.

## Results

**Fig. 1.**
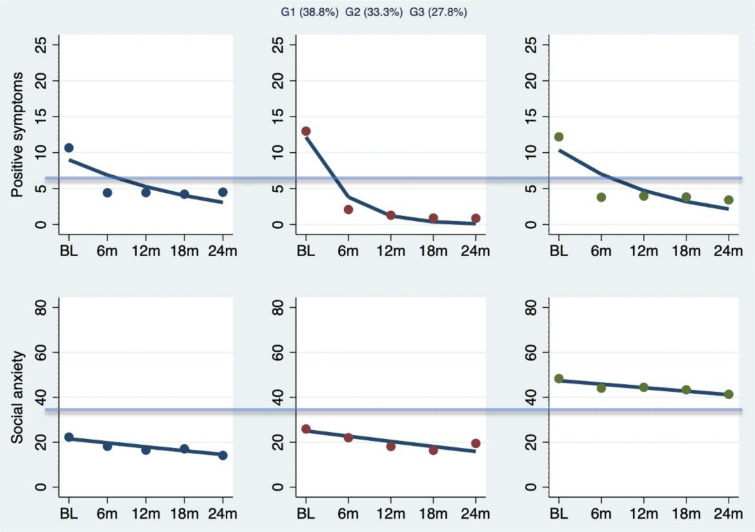
Group-based trajectory modeling for positive symptoms and social anxiety changes. *Notes.* BL = baseline, 6/12/18/24m = 6/12/18/24-month follow-up. *n*_G1_ = 290 (38.8%), *n*_G2_ = 276 (33.3%), *n*_G3_ = 198 (27.8%). Cut-off lines indicate moderate-to-severe symptom thresholds.

We selected the three-group trajectory model because the groups appeared theoretically meaningful and the criteria for parameters assessing model fitness were met, indicating that the groups were stable and distinct from one another. Figure 1 displays the trajectories in positive symptoms and social anxiety of each group across time, with cut-off lines indicating moderate to high severity thresholds. The three groups corresponded to individuals who exhibited consistently low levels of social anxiety and moderate (group 1, 38.8%) to large improvements in positive symptoms (group 2, 33.3%); and individuals who exhibited high levels of social anxiety severity despite moderate recovery in positive symptoms (group 3, 27.8%). In addition, while all three groups observed a moderate to large reduction of positive symptoms (see online Supplementary Table S2), only group 3 showed continuity of social anxiety, with levels remaining above the cut-off for high severity at the final follow-up (SIAS score = 36; see online Supplementary Table S3).

The three derived trajectory groups (see online Supplementary Table S4) did not differ significantly by race or mean parental education levels (see Table 2). The groups differed significantly on age, with group 3 exhibiting the highest mean age (19.2), followed by group 2 (18.6) and group 1 (17.9) (*F* = 5.55, df = 2, *p* = 0.004). Group 3 also had the highest representation of females (54.0%), followed by group 2 (42.0%) and group 1 (36.2%) (χ^2^ = 15.42, df = 2, *p* < 0.001).

**Table 2.** Baseline characteristics and outcome at 24 months across three trajectory groups

	Group 1 *n* = 290	Group 2 *n* = 276	Group 3 *n* = 198	Test statistic χ^2^
	Frequency (%)			
Sex (%female)	105 (36.2)	116 (42.0)	107 (54.0)	15.417***
Race (%Caucasian)	174 (60.2)	150 (54.3)	113 (57.1)	1.985
	Mean (*s.d.*)			*F*
Age (years)	17.94 (*3.97*)	18.58 (*4.33*)	19.22 (*4.37*)	5.55**
Parental education level	6.38 (*1.44*)	6.26 (*1.42*)	6.24 (*1.45*)	0.66
24-month global functioning	61.11 (*13.19*)	60.24 (*15.42*)	54.82 (*12.87*)	6.16**

**p < 0.01; ***p < 0.001.

In terms of global functional outcomes, individuals in group 3 exhibited significantly lower levels of global functioning ability at 24-months (*F* = 6.16, df = 2, *p* = 0.002; see Table 2). In examining the baseline predictors of group 3 membership (i.e. individuals with consistently high levels of social anxiety despite moderate improvement in positive symptoms; see online Supplementary Table S5) *v.* the other two groups (1 and 2) collapsed together, logistic regression models showed significant main effects of both genetic risk and environmental stress on predicting membership in the trajectory group showing a consistently high level of social anxiety over time (see Table 3a). However, no significant interaction was found between the PRS and environmental stress. The main effects remained significant after controlling for sex (see Table 3b) and age (see Table 3c) respectively.

**Table 3.** Binary logistic regression analysis for variables predicting group 3 membership (consistently high levels of social anxiety over 2 years)

(a) Regression model predicting group 3 (high social anxiety) membership without covariates				
Predictors	B	s.e.	*p* value	OR
Environmental stress (E)	0.23	0.06	<0.001	1.26
Genetic risk (G)	0.06	0.03	0.038	1.06
G × E interaction	0.01	0.01	0.366	1.01

(b) Regression model predicting group 3 (high social anxiety) membership controlling for sex				
Predictors	B	s.e.	*p* value	OR
Environmental stress (E)	0.20	0.06	<0.001	1.23
Genetic risk (G)	0.06	0.03	0.027	1.06
G × E interaction	0.01	0.01	0.377	1.01
Sex	0.49	0.23	0.028	1.64

(c) Regression model predicting group 3 (high social anxiety) membership controlling for age				
Predictors	B	s.e.	*p* value	OR
Environmental stress (E)	0.21	0.06	<0.001	1.23
Genetic risk (G)	0.06	0.03	0.037	1.06
G × E interaction	0.01	0.01	0.298	1.01
Age	0.03	0.03	0.175	1.04

Model Statistics: χ^2^_a_ = 25.68***, χ^2^_b_ = 30.51***, χ^2^_c_ = 27.50***. ***p < 0.001.

The percentage of participants who converted to psychosis during the study period (i.e. 24 months following the baseline assessment) did not differ significantly between group 3 (13.6%) and the other two groups (13.5%) (χ^2^ < 0.01, df = 1, *p* = 0.967). In other words, while the converters were included in the trajectory analysis, the status of conversion to psychosis did not influence the forming of trajectory subgroups. Indeed, among those who converted to psychosis, the majority converted (and were dropped out of the analysis) before the 6-month (56.3%) or 12-month (82.5%) follow-up timepoint. Given that most of the data were not captured at follow-up assessments, the converters primarily influenced the analysis as an intercept at baseline, but did not contribute as much to the calculation of slopes.

## Discussion

This study identified a distinctive subgroup of CHR-P cases who maintained high levels of social anxiety over 2 years, despite moderate recovery in positive symptom severity. The presence of this group was determined using a discovery-oriented technique sensitive to detecting covariant patterns of change over time on multiple indicators (in this case, positive symptoms and social anxiety) simultaneously. Further evidence of the meaningful distinction between this trajectory and the others observed came from analyses comparing the trajectory groups on long-term outcomes and risk factors for schizophrenia spectrum disorders. Membership in the group in whom social anxiety symptoms remained in the severe range over time was predicted by higher levels of polygenic risk for schizophrenia and environmental stress and was associated with poorer global long-term outcome (i.e. GAF). Taken together, the validation analyses showing differences in etiological influences and functional outcomes confirm the importance of identifying a CHR-P subgroup with stable patterns of social anxiety symptoms over time.

Environmental risk in this study was characterized by stressors an individual may be exposed to during different time periods of their life. Prior work has shown that both early life trauma and subsequent adverse life events are associated with the development of psychotic illnesses (Mayo et al., 2017). Prior work further suggested that stressors at different life stages may synergize with one another, leading to poor prognosis and functional outcomes. According to the stress-sensitization model (Harkness, Hayden, & Lopez-Duran, 2015), biological vulnerability may increase the likelihood of an individual experiencing psychiatric symptoms following early life stressors such as childhood trauma. At the early stage of psychopathology, vulnerability further increases, rendering an individual more susceptible to subsequent major stressors such adverse life events (Pruessner, Cullen, Aas, & Walker, 2017). Eventually, stress exposure may lead to greater difficulty coping with daily life stressors in the CHR-P population (Trotman et al., 2014). The composite score created in this study encapsulates a broad range of life stressors that are relevant to the symptom development and functional outcomes, providing a novel and succinct way of characterizing environmental risk factors for the CHR-P individuals.

Membership in the trajectory group that displayed continuing high levels of social anxiety was also predicted by higher PRSs for schizophrenia. This pattern is consistent with prior work showing social anxiety as a behavioral feature elevated among individuals at genetic risk for schizophrenia [such as adoptees with parents with schizophrenia (Kety, 1988)] and with the inclusion of social anxiety in the diagnostic criteria for schizotypal personality disorder, which is part of the inherited spectrum of disorders associated with schizophrenia (Ettinger, Meyhofer, Steffens, Wagner, & Koutsouleris, 2014). In CHR-P individuals, variation in polygenic risk, although predictive of psychosis conversion on its own, does not contribute uniquely to conversion risk over and above other clinical, cognitive, and demographic predictors (Perkins et al., 2020).

While both genetic risk and environmental stress were found to independently predict stable patterns of co-occurring social anxiety over time, no significant interactions were observed between these two factors of etiological influence. This pattern is in line with existing literature that has found no interaction between familial risk (i.e. parental psychotic psychopathology) and trauma in contributing the development and persistence of subclinical psychosis (Wigman et al., 2012). This is further supported by recent work suggesting that trauma history may contribute to psychosis in adulthood independent of genetic vulnerability (Bernardo et al., 2017; Fusar-Poli et al., 2017; Husted, Ahmed, Chow, Brzustowicz, & Bassett, 2010). Taken together, our findings recapitulate the importance of teaching adaptive coping strategies as a treatment option in reducing environmental stress and alleviating adverse outcomes (Armando et al., 2018).

While cross-sectional research has linked elevated social anxiety with poor functional outcomes in the CHR-P individuals (Kuhney et al., 2021), this study is the first in detecting a unique subgroup with sustained social anxiety over time that implicates impaired global functioning regardless of the positive symptom recovery. Group-based multi-trajectory modeling allows for a holistic representation of the concurrent clinical course between positive symptoms and social anxiety. Our research adds to the cross-sectional findings that are limited to CHR individuals with persistent or progressive symptoms (Kuhney et al., 2021). The unique signature of sustained social anxiety agrees with prior research on the association between depressive symptom recovery and global functioning improvement in the CHR individuals (Deng et al., 2021), highlighting the importance of considering co-occurring affective symptoms in managing functioning deficits. In particular, social anxiety, even with remitting positive symptoms, remains a crucial intervention target essential to functional improvement. In addition, the differentiation between situational anxiety and persistent or sustained social anxiety that resembles schizotypal personality traits provides important treatment implications. While psychosocial interventions (e.g. cognitive behavioral therapy) may be sufficient in treating social anxiety of an episodic nature, more extensive treatments (such as metacognitive therapy designed to increase an individual's level of insight) are potentially beneficial to better address sustained anxiety in the CHR-P population.

The group-based multi-trajectory analysis not only yielded a distinct subgroup of CHR-P individuals characterized by sustained social anxiety, but also captured two groups with trajectories implying remission of anxiety (below the clinical threshold). While these two groups did not differ on social anxiety trajectory, they showed differential rates of positive symptom recovery (with group 1 showing a gradual decline and group 2 seeing a sharp decrease in positive symptoms). However, these two groups did not differ on global functional outcomes at 24 months, and showed no differential relations to either genetic or environmental risk factors. That the two groups did not differ from each other in outcome or etiologic variables could imply that they do not meaningfully separate into different clusters (i.e. the trajectory differences in degree of positive symptom recovery could be incidental). While it has been theorized that situational anxiety is likely to covary with positive symptoms, as the two symptom dimensions share underlying mechanisms such as an elevated attention to threat, this hypothesis was only partially supported by the findings. Between the two groups with remitting anxiety trajectories, positive symptoms decreased by varying degrees despite the similar amount of decline in social anxiety. This highlights that positive symptom severity can still remain at a moderate level without anxiety being present. Further work is required to better understand the covarying symptoms in the CHR-P population, especially if positive symptoms are driving the social anxiety, or vice versa.

There are a few notable limitations. While the PRS provides an approximate representation of the degree of genetic risk for schizophrenia among the CHR-P population, the interaction between genetic and environmental risks may be better captured by examining specific genetic polymorphisms instead of the combined PRS. Therefore, the ability to rule out the joint effects of genetic and environmental risk factors is limited in the current study. Further, given that the conversion to psychosis occurred most frequently in the first 8 months after baseline, contributing to the sharp decline in symptoms from baseline to 6-month follow-up, the current study design (with follow-up assessments every 6 months) may be too coarse to capture the differential non-decline of positive symptoms manifested among the converters. Future research may benefit from a more fine-grained measurement of symptom trajectories by adding more frequent follow-up assessments and considering experience sampling methods, especially at the early stage of risk syndrome development. In addition, while the GAF provides a holistic representation of global functional outcomes encompassing both symptom distress and functioning levels, it is influenced by symptom severity (such as social anxiety), therefore limiting its utility solely as a functional outcome measure. Future research may continue to expand this line of work by considering social functioning and symptom distress as separate outcome variables.

To conclude, the present study identified a distinctive CHR-P subgroup with stable patterns of social anxiety symptoms over time. This subgroup with sustained social anxiety was associated with genetic and environmental risk factors for schizophrenia as well as poorer global long-term outcome, regardless of the positive symptom recovery. These findings highlight the importance of considering co-occurred social anxiety in early interventions for psychosis.

## Supplementary material

For supplementary material accompanying this paper visit https://doi.org/10.1017/S0033291722000277. click here to view supplementary material

## References

[ref1] Addington, J., Liu, L., Buchy, L., Cadenhead, K. S., Cannon, T. D., Cornblatt, B. A., … McGlashan, T. H. (2015). North American prodrome longitudinal study (NAPLS 2): The prodromal symptoms. The Journal of Nervous and Mental Disease, 203(5), 328–335. doi: 10.1097/NMD.0000000000000290 25919383 PMC4417745

[ref2] Addington, J., Piskulic, D., Liu, L., Lockwood, J., Cadenhead, K. S., Cannon, T. D., … Woods, S. W. (2017). Comorbid diagnoses for youth at clinical high risk of psychosis. Schizophrenia Research, 190, 90–95. doi: 10.1016/j.schres.2017.03.043 28372906 PMC5731830

[ref3] Addington, J., Stowkowy, J., Cadenhead, K. S., Cornblatt, B. A., McGlashan, T. H., Perkins, D. O., … Woods, S. W. (2013). Early traumatic experiences in those at clinical high risk for psychosis. Early Intervention in Psychiatry, 7(3), 300–305. doi: 10.1111/eip.12020 23343384 PMC3754436

[ref4] Allswede, D. M., Addington, J., Bearden, C. E., Cadenhead, K. S., Cornblatt, B. A., Mathalon, D. H., … Cannon, T. D. (2019). Characterizing covariant trajectories of individuals at clinical high risk for psychosis across symptomatic and functional domains. American Journal of Psychiatry, 177(2), 164–171. doi: 10.1176/appi.ajp.2019.18111290 31509005 PMC7002249

[ref5] Armando, M., Sandini, C., Chambaz, M., Schaer, M., Schneider, M., & Eliez, S. (2018). Coping strategies mediate the effect of stressful life events on schizotypal traits and psychotic symptoms in 22q11. 2 deletion syndrome. Schizophrenia Bulletin, 44(Suppl_2), S525–S535. doi: 10.1093/schbul/sby025 29548017 PMC6188528

[ref6] Bernardo, M., Bioque, M., Cabrera, B., Lobo, A., Gonzalez-Pinto, A., Pina, L., … Mas, S. (2017). Modelling gene-environment interaction in first episodes of psychosis. Schizophrenia Research, 189, 181–189. doi: 10.1016/j.schres.2017.01.058 28179063

[ref7] Brantley, P. J., Waggoner, C. D., Jones, G. N., & Rappaport, N. B. (1987). A Daily Stress Inventory: Development, reliability, and validity. Journal of Behavioral Medicine, 10(1), 61–74. doi: 10.1007/BF00845128 3586002

[ref8] Chang, W. C., Ng, C. M., Chan, K. N., Lee, H. C., Chan, S. I., Chiu, S. S., … Chen, E. Y. H. (2021). Psychiatric comorbidity in individuals at-risk for psychosis: Relationships with symptoms, cognition and psychosocial functioning. Early Intervention in Psychiatry, 15(3), 616–623. doi: 10.1111/eip.12992 32441490

[ref9] Deng, W., Addington, J., Bearden, C. E., Cadenhead, K. S., Cornblatt, B. A., Mathalon, D. H., … Cannon, T. (2021). Depression predicts global functional outcomes in individuals at clinical high risk for psychosis. Psychiatric Research and Clinical Practice, 3(4), 163–171. doi: 10.1176/appi.prcp.20210023 36101655 PMC9175802

[ref10] Dohrenwend, B. S., Krasnoff, L., Askenasy, A. R., & Dohrenwend, B. P. (1978). Exemplification of a method for scaling life events: The Peri Life Events Scale. Journal of Health and Social Behavior, 19(2), 205–229. Retrieved from https://www.ncbi.nlm.nih.gov/pubmed/681735. 681735

[ref11] Ettinger, U., Meyhofer, I., Steffens, M., Wagner, M., & Koutsouleris, N. (2014). Genetics, cognition, and neurobiology of schizotypal personality: A review of the overlap with schizophrenia. Frontiers in Psychiatry, 5, 18. doi: 10.3389/fpsyt.2014.00018 24600411 PMC3931123

[ref12] Fergus, T. A., Valentiner, D. P., Kim, H. S., & McGrath, P. B. (2014). The Social Interaction Anxiety Scale (SIAS) and the Social Phobia Scale (SPS): A comparison of two short-form versions. Psychological Assessment, 26(4), 1281–1291. doi: 10.1037/a0037313 24978134

[ref13] Fonseca-Pedrero, E., Debbane, M., Ortuno-Sierra, J., Chan, R. C. K., Cicero, D. C., Zhang, L. C., … Jablensky, A. (2018). The structure of schizotypal personality traits: A cross-national study. Psychological Medicine, 48(3), 451–462. doi: 10.1017/S0033291717001829 28712364

[ref14] Freeman, D., & Fowler, D. (2009). Routes to psychotic symptoms: Trauma, anxiety and psychosis-like experiences. Psychiatry Research, 169(2), 107–112. doi: 10.1016/j.psychres.2008.07.009 19700201 PMC2748122

[ref15] Fulford, D., Niendam, T. A., Floyd, E. G., Carter, C. S., Mathalon, D. H., Vinogradov, S., … Loewy, R. L. (2013). Symptom dimensions and functional impairment in early psychosis: More to the story than just negative symptoms. Schizophrenia Research, 147(1), 125–131. doi: 10.1016/j.schres.2013.03.024 23587696 PMC3663589

[ref16] Fusar-Poli, P., Tantardini, M., De Simone, S., Ramella-Cravaro, V., Oliver, D., Kingdon, J., … McGuire, P. (2017). Deconstructing vulnerability for psychosis: Meta-analysis of environmental risk factors for psychosis in subjects at ultra high-risk. European Psychiatry, 40, 65–75. doi: 10.1016/j.eurpsy.2016.09.003 27992836

[ref17] Harkness, K. L., Hayden, E. P., & Lopez-Duran, N. L. (2015). Stress sensitivity and stress sensitization in psychopathology: An introduction to the special section. Journal of Abnormal Psychology, 124(1), 1–3. doi: 10.1037/abn0000041 25688427

[ref18] Husted, J. A., Ahmed, R., Chow, E. W., Brzustowicz, L. M., & Bassett, A. S. (2010). Childhood trauma and genetic factors in familial schizophrenia associated with the NOS1AP gene. Schizophrenia Research, 121(1–3), 187–192. doi: 10.1016/j.schres.2010.05.021 20541371 PMC3127865

[ref19] Janssen, I., Krabbendam, L., Bak, M., Hanssen, M., Vollebergh, W., de Graaf, R., & van Os, J. (2004). Childhood abuse as a risk factor for psychotic experiences. Acta Psychiatrica Scandinavica, 109(1), 38–45. doi: 10.1046/j.0001-690x.2003.00217.x 14674957

[ref20] Jones, B. L., & Nagin, D. S. (2013). A note on a Stata plugin for estimating group-based trajectory models. Sociological Methods & Research, 42(4), 608–613. doi: 10.1177/0049124113503141

[ref21] Kety, S. S. (1988). Schizophrenic illness in the families of schizophrenic adoptees: Findings from the Danish national sample. Schizophrenia Bulletin, 14(2), 217–222. doi: 10.1093/schbul/14.2.217 3201179

[ref22] Kuhney, F. S., Damme, K. S. F., Pelletier-Baldelli, A., Chun, C., Ellman, L. M., Schiffman, J., & Mittal, V. A. (2021). Prevalence and functional consequences of social anxiety in individuals at clinical high-risk for psychosis: Perspective from a community sample comparison. Schizophrenia Bulletin Open, 2(1), sgab025. doi: 10.1093/schizbullopen/sgab025 34308353 PMC8295730

[ref23] Kupper, N., & Denollet, J. (2012). Social anxiety in the general population: Introducing abbreviated versions of SIAS and SPS. Journal of Affective Disorders, 136(1–2), 90–98. doi: 10.1016/j.jad.2011.08.014 21903277

[ref24] Lam, M., Awasthi, S., Watson, H. J., Goldstein, J., Panagiotaropoulou, G., Trubetskoy, V., … Ripke, S. (2020). RICOPILI: Rapid Imputation for COnsortias PIpeLIne. Bioinformatics (Oxford, England), 36(3), 930–933. doi: 10.1093/bioinformatics/btz633 31393554 PMC7868045

[ref25] Manichaikul, A., Mychaleckyj, J. C., Rich, S. S., Daly, K., Sale, M., & Chen, W. M. (2010). Robust relationship inference in genome-wide association studies. Bioinformatics (Oxford, England), 26(22), 2867–2873. doi: 10.1093/bioinformatics/btq559 20926424 PMC3025716

[ref26] Mayo, D., Corey, S., Kelly, L. H., Yohannes, S., Youngquist, A. L., Stuart, B. K., … Loewy, R. L. (2017). The role of trauma and stressful life events among individuals at clinical high risk for psychosis: A review. Frontiers in Psychiatry, 8, 55. doi: 10.3389/fpsyt.2017.00055 28473776 PMC5397482

[ref27] McAusland, L., Buchy, L., Cadenhead, K. S., Cannon, T. D., Cornblatt, B. A., Heinssen, R., … Addington, J. (2017). Anxiety in youth at clinical high risk for psychosis. Early Intervention Psychiatry, 11(6), 480–487. doi: 10.1111/eip.12274 PMC4912451 26456932

[ref28] McGlashan, T. H., Walsh, B., & Woods, S. (2010). The psychosis-risk syndrome: Handbook for diagnosis and follow-up. New York, NY: Oxford University Press. Retrieved from http://www.loc.gov/catdir/enhancements/fy1013/2009045758-b.html.

[ref29] Michail, M., & Birchwood, M. (2009). Social anxiety disorder in first-episode psychosis: Incidence, phenomenology and relationship with paranoia. British Journal of Psychiatry, 195(3), 234–241. doi: 10.1192/bjp.bp.108.053124 19721113

[ref30] Osborne, K. J., & Mittal, V. A. (2019). External validation and extension of the NAPLS-2 and SIPS-RC personalized risk calculators in an independent clinical high-risk sample. Psychiatry Research, 279, 9–14. doi: 10.1016/j.psychres.2019.06.034 31279247 PMC6713610

[ref31] Pedersen, G., & Karterud, S. (2012). The symptom and function dimensions of the Global Assessment of Functioning (GAF) scale. Comprehensive Psychiatry, 53(3), 292–298. doi: 10.1016/j.comppsych.2011.04.007 21632038

[ref32] Perkins, D. O., Olde Loohuis, L., Barbee, J., Ford, J., Jeffries, C. D., Addington, J., … Woods, S. W. (2020). Polygenic risk score contribution to psychosis prediction in a target population of persons at clinical high risk. American Journal of Psychiatry, 177(2), 155–163. doi: 10.1176/appi.ajp.2019.18060721 31711302 PMC7202227

[ref33] Peters, L. (2000). Discriminant validity of the Social Phobia and Anxiety Inventory (SPAI), the Social Phobia Scale (SPS) and the Social Interaction Anxiety Scale (SIAS). Behaviour Research and Therapy, 38(9), 943–950. doi: 10.1016/s0005-7967(99)00131-x 10957828

[ref34] Pruessner, M., Cullen, A. E., Aas, M., & Walker, E. F. (2017). The neural diathesis-stress model of schizophrenia revisited: An update on recent findings considering illness stage and neurobiological and methodological complexities. Neuroscience and Biobehavioral Reviews, 73, 191–218. doi: 10.1016/j.neubiorev.2016.12.013 27993603

[ref35] Rosell, D. R., Futterman, S. E., McMaster, A., & Siever, L. J. (2014). Schizotypal personality disorder: A current review. Current Psychiatry Reports, 16(7), 452. doi: 10.1007/s11920-014-0452-1 24828284 PMC4182925

[ref36] Salokangas, R. K., Dingemans, P., Heinimaa, M., Svirskis, T., Luutonen, S., Hietala, J., … Klosterkotter, J. (2013). Prediction of psychosis in clinical high-risk patients by the Schizotypal Personality Questionnaire: Results of the EPOS project. European Psychiatry, 28(8), 469–475. doi: 10.1016/j.eurpsy.2013.01.001 23394823

[ref37] Schizophrenia Working Group of the Psychiatric Genomics Consortium (2014). Biological insights from 108 schizophrenia-associated genetic loci. Nature, 511(7510), 421–427. doi: 10.1038/nature13595 25056061 PMC4112379

[ref38] Schwartz, R. C. (2007). Concurrent validity of the Global Assessment of Functioning Scale for clients with schizophrenia. Psychological Reports, 100(2), 571–574. doi: 10.2466/pr0.100.2.571-574 17564234

[ref39] Startup, M., Jackson, M. C., & Bendix, S. (2002). The concurrent validity of the Global Assessment of Functioning (GAF). British Journal of Clinical Psychology, 41(Pt 4), 417–422. doi: 10.1348/014466502760387533 12437796

[ref40] Stowkowy, J., Liu, L., Cadenhead, K. S., Cannon, T. D., Cornblatt, B. A., Mcglashan, T. H., … Addington, J. (2016). Early traumatic experiences, perceived discrimination and conversion to psychosis in those at clinical high risk for psychosis. Social Psychiatry and Psychiatric Epidemiology, 51(4), 497–503. doi: 10.1007/s00127-016-1182-y 26851943 PMC7012367

[ref41] Trotman, H. D., Holtzman, C. W., Walker, E. F., Addington, J. M., Bearden, C. E., Cadenhead, K. S., … McGlashan, T. H. (2014). Stress exposure and sensitivity in the clinical high-risk syndrome: Initial findings from the North American Prodrome Longitudinal Study (NAPLS). Schizophrenia Research, 160(1–3), 104–109. doi: 10.1016/j.schres.2014.09.017 25443665 PMC4593703

[ref42] Wigman, J. T., van Winkel, R., Ormel, J., Verhulst, F. C., van Os, J., & Vollebergh, W. A. (2012). Early trauma and familial risk in the development of the extended psychosis phenotype in adolescence. Acta Psychiatrica Scandinavica, 126(4), 266–273. doi: 10.1111/j.1600-0447.2012.01857.x 22486536

[ref43] Woods, S. W., Walsh, B. C., Powers, A. R., & McGlashan, T. H. (2019). Reliability, validity, epidemiology, and cultural variation of the Structured Interview for Psychosis-risk Syndromes (SIPS) and the Scale Of Psychosis-risk Symptoms (SOPS). In H. Li, D. Shapiro, & L. Seidman (Eds.), Handbook of attenuated psychosis syndrome across cultures (pp. 85–113). Cham: Springer. doi: 10.1007/978-3-030-17336-4_5

