# Supplementary material for: Characterizing sustained social anxiety in individuals at clinical high risk for psychosis: trajectory, risk factors, and functional outcomes
Source: Psychol Med. 2022 Feb 11;53(8):3644–51. doi: 10.1017/S0033291722000277 (PMC10277760; doi:10.1017/S0033291722000277)
Supplement: Supplementary file 1 [file S0033291722000277sup001.docx]

Supplemental Table 1.

*Positive Symptoms and Social Anxiety Variable Distribution at Baseline, 6-, 12-, 18- and 24-month*

*(a) Positive Symptoms*

|  | Baseline | 6-month | 12-month | 18-month | 24-month |
| --- | --- | --- | --- | --- | --- |
| M | 11.91 | 3.52 | 3.51 | 3.41 | 3.42 |
| SD | 3.818 | 2.938 | 2.939 | 2.816 | 2.786 |

*(b) Social Anxiety*

|  | Baseline | 6-month | 12-month | 18-month | 24-month |
| --- | --- | --- | --- | --- | --- |
| M | 30.8 | 26.53 | 25.12 | 25.66 | 24.07 |
| SD | 17.406 | 16.793 | 17.517 | 17.176 | 16.995 |

Supplemental Table 2.

*Maximum Likelihood Estimates for Positive Symptoms Trajectory*

| Group | Parameter | Estimate | Standard Error | T for H0: Parameter=0 | Prob > \|T\| |
| --- | --- | --- | --- | --- | --- |
| 1 | Intercept | 2.466 | 0.032 | 76.638 | <.001 |
|  | Linear | -0.268 | 0.013 | -20.326 | <.001 |
| 2 | Intercept | 3.659 | 0.063 | 58.543 | <.001 |
|  | Linear | -1.160 | 0.049 | -23.827 | <.001 |
| 3 | Intercept | 2.730 | 0.041 | 66.721 | <.001 |
|  | Linear | -0.393 | 0.022 | -18.104 | <.001 |

*Notes:* Model: Zero Inflated Poisson (zip)

Supplemental Table 3.

*Maximum Likelihood Estimates for Social Anxiety Trajectory*

| Group | Parameter | Estimate | Standard Error | T for H0: Parameter=0 | Prob > \|T\| |
| --- | --- | --- | --- | --- | --- |
| 1 | Intercept | 23.191 | 1.081 | 21.460 | <.001 |
|  | Linear | -1.931 | 0.341 | -5.672 | <.001 |
| 2 | Intercept | 27.352 | 1.598 | 17.122 | <.001 |
|  | Linear | -2.445 | 0.519 | -4.709 | <.001 |
| 3 | Intercept | 49.056 | 1.281 | 38.282 | <.001 |
|  | Linear | -1.577 | 0.401 | -3.930 | <.001 |

*Notes:* Model: Censored Normal (cnorm)

Supplemental Table 4.

*Absolute Model Fit Statistics*

| Group | Parameter | Estimate | Standard Error | T for H0: Parameter=0 | Prob > \|T\| | average posterior probabilities | odds of correct classification |
| --- | --- | --- | --- | --- | --- | --- | --- |
| 1 | (%) | 38.824 | 2.472 | 15.705 | <.001 | 0.848 | 9.064 |
| 2 | (%) | 33.334 | 2.572 | 12.959 | <.001 | 0.788 | 7.224 |
| 3 | (%) | 27.842 | 2.514 | 11.074 | <.001 | 0.875 | 18.224 |

*Note:* Bayesian information criterion (BIC) for the total number of observations=-14790.37 (N=4339); BIC for the total number of participants =-14776.83 (N=713); Akaike information criterion (AIC) =-14742.56.

Extra groups were added (2-, 3-, and 4-groups) until the best fitting model (with the highest/least negative value of BIC and AIC) was established.

Supplemental Table 5.

*SCID-based Social Phobia Diagnostic Status by Group (Number of Participants Diagnosed with Social Phobia (%)*)

|  | Baseline | 12-month | 24-month |
| --- | --- | --- | --- |
| Group 1 | 19 (6.7%) | 7 (3.8%) | 8 (6.2%) |
| Group 2 | 34 (12.9%) | 8 (8.1%) | 5 (9.8%) |
| Group 3 | 54 (27.4%) | 33 (27.3%) | 20 (23.3%) |
